# The Role of Snx41-Based Pexophagy in Magnaporthe Development

**DOI:** 10.1371/journal.pone.0079128

**Published:** 2013-11-01

**Authors:** Yizhen Deng, Ziwei Qu, Naweed I. Naqvi

**Affiliations:** 1 Temasek Life Sciences Laboratory, and Department of Biological Sciences, National University of Singapore, Singapore, Singapore; 2 School of Biological Sciences, Nanyang Technological University, Singapore, Singapore; California Department of Public Health, United States of America

## Abstract

Pexophagy, the degradation of peroxisomes via selective autophagy, depends on Atg20/Snx42 function in *Saccharomyces cerevisiae*. Besides its role in selective autophagy, Atg20/Snx42 is also involved in an autophagy-independent endosomal retrieval trafficking, in cooperation with two other sorting nexins, Snx41 and Snx4. Recently, we reported that the sorting nexin MoSnx41, which showed high sequence similarity to yeast Snx41 and Snx42/Atg20 proteins, regulates the gamma-glutamyl cycle and GSH production and is essential for conidiation and pathogenicity in *Magnaporthe oryzae*. Pexophagy was also found to be defective in *Mosnx41*Δ mutant. These findings indicate that MoSnx41 likely serves combined functions of Snx42/Atg20 and Snx41 in *M. oryzae*.. In this study, we performed complementation analyses and demonstrate that MoSnx41 alone serves the dual function of protein sorting (ScSnx41) and pexophagy (ScSnx42/Atg20). To study the potential biological function of pexophagy in fungal pathogenic life cycle, we created deletion mutants of potential pexophagy-specific genes, and characterized them in terms of pexophagy, conidiation and pathogenesis. We identified Pex14 as an essential protein for pexophagy in *M. oryzae*. Overall, our results show that pexophagy *per se* is not essential for asexual development or virulence in *M. oryzae*.

## Introduction

Autophagy is a bulk degradation process conserved in all eukaryotes. Besides non-selective autophagy, there are several subtypes of selective autophagy, such as the Cvt pathway [1], mitophagy [2,3] and pexophagy [4,5]. 

Peroxisomes are unit membrane-bound organelles, containing peroxidase and catalase activities and contribute to several lipid metabolic pathways and redox homeostasis in eukaryotic cells [6,7]. So far 32 *PEX* genes have been identified and the encoded peroxins are indispensable for peroxisome biogenesis [8,9]. Peroxisome biogenesis can be induced by the presence of fatty acids, and degradation of the organelle in vacuoles (pexophagy) is in response to glucose [5,10,11]. 

The role of peroxisomal metabolism and pexophagy in fungal pathogenicity is not well understood. Peroxisomal biogenesis protein Pex6 is required for pathogenicity in *Colletotrichum orbiculare*, the fungal pathogen causing cucumber anthracnose [12,13], and in the rice-blast fungus *M. oryzae* [14]. On the other hand, pexophagy was found to be required for host invasion by *C. orbiculare* [15]. The *M. oryzae* genome contains a subset of *ATG* and *PEX* genes, whose orthologs in yeasts are dispensable for non-selective autophagy but serve as critical pexophagy regulators. Thus, such specific *ATG* or *PEX* candidate genes are well-suited for studying the biological function of pexophagy in fungal pathogenesis. Atg26 was reported to be essential for pexophagy in Podospora, Pichia [16,17] and in *C. orbiculare* [18]. It remains unclear whether Atg26 is essential for pexophagy in *M. oryzae*, although an *M. oryzae atg26*Δ mutant has been shown to be pathogenic in a recent genome wide study [19]. Atg20, an autophagy protein, was shown to be essential for the Cvt (cytosol-to-vacuole targeting) pathway and pexophagy in yeast [20]. However, previous screening with yeast orthologs showed that the *ATG* genes specifically involved in the Cvt pathway and/or pexophagy were poorly conserved in filamentous fungi, including *M. oryzae* [21]. Based on this study, it is believed that there is no Atg20/Snx42 like protein in *M. oryzae*. In *Hansenula polymorpha*, a peroxisomal membrane-anchoring Peroxin 14 (Pex14) was implicated in both peroxisome biogenesis and pexophagy [22,23]. Recently, we reported an *M. oryzae* sorting nexin, Snx41, to be orthologous in sequence to both Snx41 and Snx42/Atg20 from *S. cerevisiae*. MoSnx41 is dispensable for non-selective autophagy, but essential for pexophagy, which is functionally similar to *S. cerevisiae* Snx42/Atg20. More importantly, it remains to be investigated whether pexophagy plays a role in *M. oryzae* conidiation and/or pathogenicity. 

In this study, we assessed the role of *ATG26* and *PEX14*, which are single-copy genes in *M. oryzae*, in pexophagy. Our results indicate that Atg26 is not required for pexophagy in *M. oryzae*. Whereas, the N-terminal cytosolic domain (amino acid 1-60) of Pex14 was found to be essential for pexophagy in *M. oryzae*. Gene complementation analysis showed that *M. oryzae* Snx41 is functionally equivalent to ScSnx42/ScAtg20 in pexophagy, while being similar to ScSnx41 in retrieval trafficking function. However, the biological defects in *snx41*Δ were more relevant to Snx41-dependent retrieval trafficking, rather than Snx42/Atg20-associated pexophagy [24]. Lastly, we demonstrate that pexophagy does not occur during *M. oryzae* conidiation. Consistent with this, pexophagy is dispensable for *M. oryzae* asexual development. 

## Results

### Pexophagy during asexual development in *M. oryzae*

**Figure 1:**
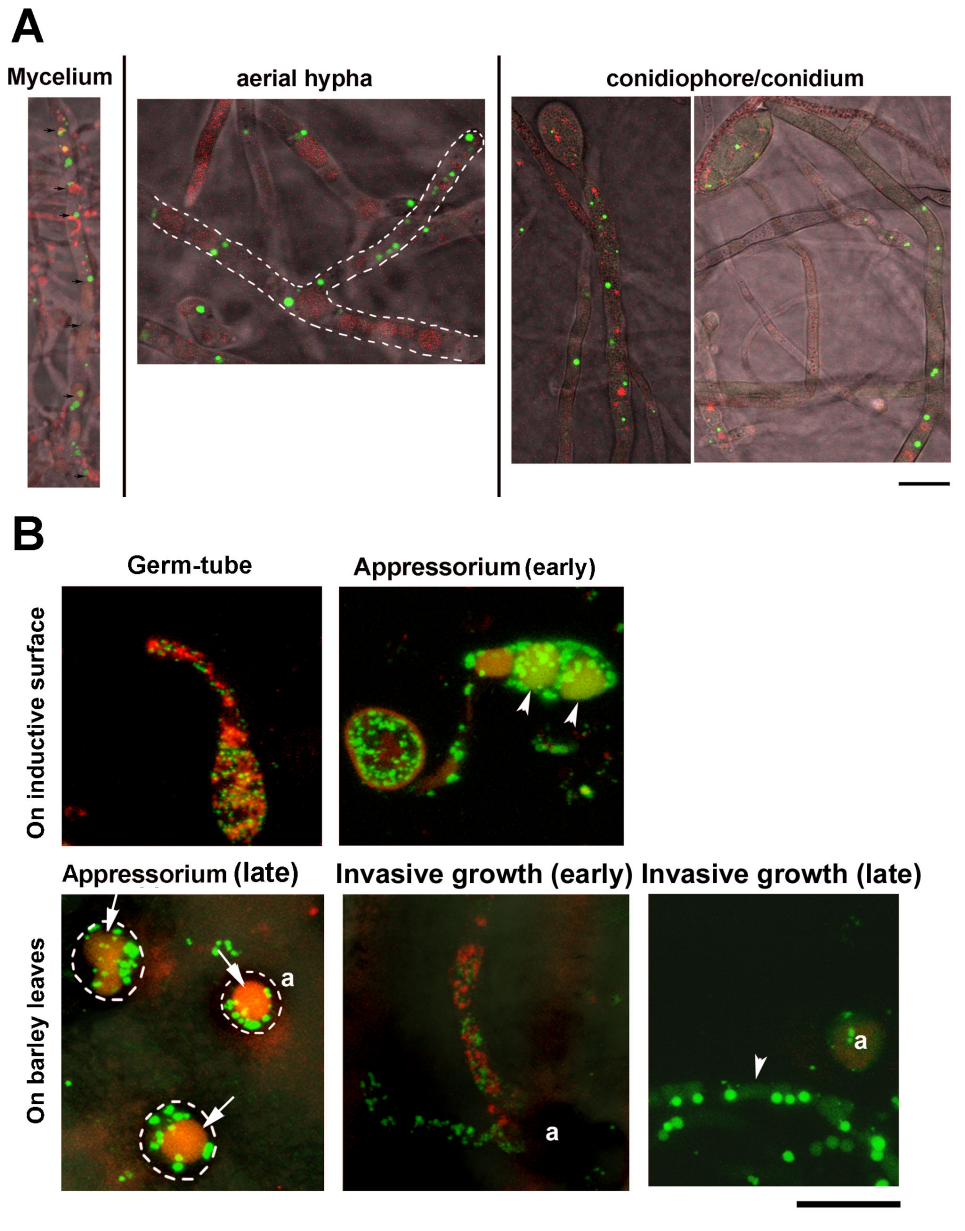
Analysis of pexophagy during *M. oryzae* conidiation and pathogenic development. (A) Confocal microscopy of conidiating cultures grown on PA medium, from *M. oryzae* GFP-SRL/RFP-Atg8 strain shows the lack of pexophagy therein during asexual development. Images shown are representative of the developmental stage depicted by the majority of vegetative mycelia, aerial hyphae or conidiophores. Dashed outline depicts aerial hyphae and its connecting mycelium. Scale bar equals 5 micron. (B) Pexophagy is naturally induced during appressorium formation and function. Conidia from the GFP-SRL/RFP-Atg8 strain were inoculated on inductive surface and visualized by confocal microscopy, at the indicated stages of germination (2-4 hpi), appressorium initiation (5-8 hpi) and appressorium maturation (14-16 hpi). For the assessment of *in* *planta* differentiation, conidia were inoculated on barley leaf explants and visualized by confocal microscopy at the indicated stages. Arrowheads denote vacuoles containing GFP-SRL (peroxisomes). Arrows denote vacuoles without GFP-SRL. Scale bar equals 10 micron.

In order to observe whether pexophagy occurs naturally during *M. oryzae* conidiogenesis and pathogenesis, the interaction between peroxisomes (labeled by GFP-SRL [14]) and autophagic vesicles (RFP-Atg8 [25]) was examined during conidia and appressoria development. Time-lapse observation of peroxisomes was performed in vegetative hyphae and conidiating cultures grown on Prune Agar (PA) medium (24 h post photo-induction), and during *in planta* development (Figure 1A and B). In the vegetative phase, peroxisomes (labeled using GFP-SRL; Peroxisome Targeting Sequence 1) frequently associated with the filamentous or spherical vacuoles that contained RFP-Atg8 (Figure 1A; left panel; n=100; *p* < 0.05). However, when conidiation was induced in response to light, peroxisomes and autophagic vesicles were distinctly separate in conidiation-related structures, including the aerial hyphae (Figure 1A; middle panel; n=100; *p* < 0.05), the conidiophore and the conidia (Figure 1A; right panel; n=100; *p* < 0.05), indicating a likely lack of pexophagy or peroxisome turnover during *M. oryzae* conidiation. We infer that pexophagy does not occur in the aerial hyphae or conidiophores in the rice-blast fungus. 

To monitor pexophagy during appressorium development, conidia were harvested and inoculated on an artificial inductive surface or on leaf explants from barley. Several relevant time points were chosen during appressorium differentiation. Pexophagy was observed in germinating conidium (6-9 hpi), mature appressorium (16-30 hpi) and invasive hyphae (96 hpi) (Figure 1B). Peroxisomes were delivered to the vacuoles in non-germinating conidial cells during appressorium initiation or during invasive growth (Figure 1B, arrowheads), indicating that pexophagy likely occurs at these stages during pathogenesis in *M. oryzae*. 

**Figure 2:**
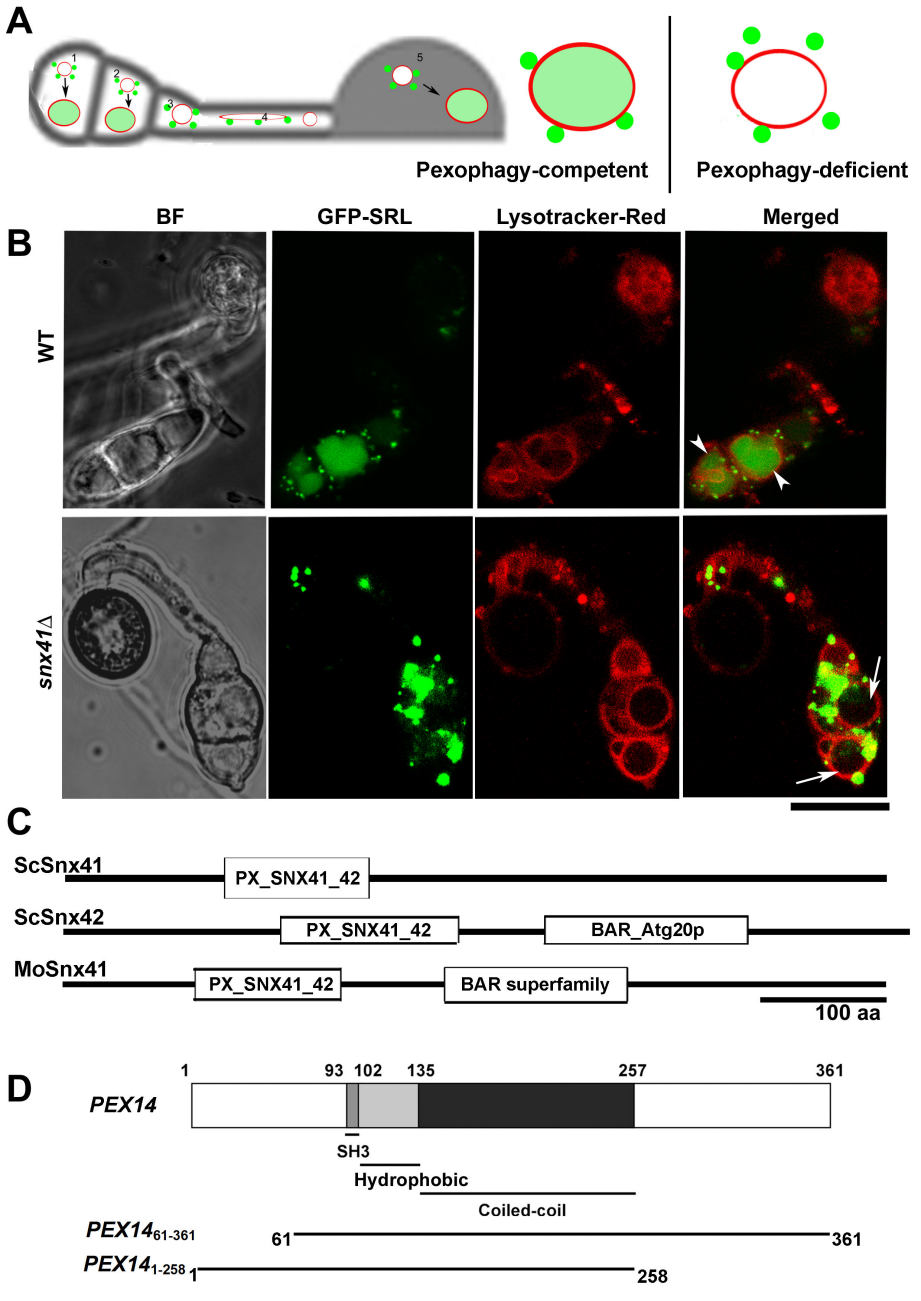
Generation of pexophagy-deficient mutants in *M. oryzae* (A) Schematic illustration of pexophagy occurring during conidial germination and appressorial development in *M. oryzae*. Vacuolar accumulation of GFP-SRL (peroxisome) was first seen in the two non-germinating cells (1 and 2) of the conidium at the early stage, whereas peroxisomes were outside the vacuoles in the germinating cell (3) and in the germ tube (4). In the newly formed appressorium (5), pexophagy was absent as peroxisomes were not delivered into the vacuoles. In mature appressoria, pexophagy occurs again and most of the peroxisomes were delivered to the lumen of the vacuoles. Right panel depicts pexophagy-competent (WT) or pexophagy-deficient situation in such cell types. Red spherical or filamentous compartments, vacuoles (stained by DND99 Lysotracker-Red); green vesicles, peroxisome labeled with GFP-SRL. (B) Pexophagy-competence and pexophagy-deficiency as illustrated by WT and *snx41*Δ mutant, respectively. Conidia were inoculated on plastic cover slip for 6-8 h, and co-stained with Lysotracker Red DND99 to label the vacuolar compartments 5 min before confocal microscopy. Arrowheads denote vacuolar GFP-SRL signal, an indication of delivery of peroxisomes to the vacuoles for pexophagy. Arrows denote vacuoles lacking GFP-SRL signal, due to blocked pexophagy. Scale bar equals 10 micron. (C) Comparison of amino sequence and domains between ScSnx41, ScSnx42 and MoSnx41. Sequence alignment and domain prediction were performed by using the BLAST program on NCBI website (http://blast.ncbi.nlm.nih.gov/Blast.cgi?PROGRAM=blastp&BLAST_PROGRAMS=blastp&PAGE_TYPE=BlastSearch&SHOW_DEFAULTS=on&LINK_LOC=blasthome). (D) Schematic drawing of the annotated domains within *M. oryzae* Pex14 polypeptide showing an SH3 domain, a hydrophobic loop and a coiled-coil domain. Precise mutant derivatives for Pex14 are depicted together with the coordinates representing amino acid residues.

We conclude that pexophagy is undetectable during *M. oryzae* conidiation, as shown in our time-lapse microscopic observation, but vacuolar degradation of peroxisomes is frequently evident during appressorial development and function. Here, we summarize and schematically illustrate the observed steps in pexophagy during conidial and appressorial development in *M. oryzae* (Figure 2A). 

### Generation and functional characterization of pexophagy-deficient mutants in *M. oryzae*

To study the biological function of pexophagy in *M. oryzae*, we first created deletion or truncated mutants of pexophagy-candidate genes detailed below. Previously, we characterized a *Mosnx41*Δ mutant, which was blocked in pexophagy and displayed loss of conidiation and pathogenicity [24]. Wild type and *snx41*Δ strains, both carrying GFP-SRL to visualize peroxisomes, were analysed to assess pexophagy-competent and pexophagy-deficient conditions, as illustrated in Figure 2B. Diffused vacuolar GFP-SRL signal (76.6 ± 3.5 %) was evident in the WT during conidial development (Figure 2B, arrowheads). In contrast, the *snx41*Δ conidia lacked vacuolar GFP-SRL signal (0 ± 0 %, *p* < 0.01 vs WT) at the comparable stage of development (Figure 2B, arrows) but instead, GFP-SRL punctae accumulated outside and/or at the periphery of the vacuoles. 

*M. oryzae* Snx41 shows extensive similarity to both Snx41 and Snx42/Atg20 from *S. cerevisiae*. Both ScSnx41 and ScSnx42/Atg20 contain Phox (PX) domain for Phosphatidylinositol 3-Phosphate (PI3P) binding and membrane anchoring (Figure 2C). BAR (Bin-Amphiphysin-Rvs) domain in conjunction with PX in ScSnx42 may be involved in membrane bending and curvature formation [26,27], while it is absent in ScSnx41 (Figure 2C). *M. oryzae* Snx41 possesses PX domain and a region with weak homology to ScSnx42/Atg20 BAR domain (Figure 2C), indicating a functional conservation with ScSnx42/Atg20. However, BLASTP analysis with *M. oryzae* Snx41 showed a higher sequence similarity overall with ScSnx41 [24]. ScSnx42/Atg20 was shown to be essential for pexophagy in *S. cerevisiae*, while ScSnx41 was not required for turnover or degradation of peroxisomes [20]. Requirement of ScSnx42/Atg20 for pexophagy depends on the BAR domain, which may facilitate membrane curvature for pexophagy-PAS formation [26] or for fusion between pexophagosomes and vacuole [27]. Both ScSnx42/Atg20 and ScSnx41 are involved in non-pexophagy functions too, e.g. endosomal sorting and retrieval trafficking [28], which again rely on BAR domain-dependent membrane bending and/or tubulation. Since ScSnx41 does not possess a BAR domain, it may depend on its partner (ScSnx42/Atg20) for such membrane bending function. *M. oryzae* Snx41 is essential for pexophagy, and is clearly involved in other vesicular trafficking events, which are unrelated to pexophagy but essential for *M. oryzae* conidiation and pathogenesis [24]. Since *M. oryzae* Snx41 contains the BAR domain, it may carry out pexophagy function in a similar fashion as its yeast homolog ScSnx42/Atg20. Also, for vesicular sorting and trafficking, as it contains a BAR motif of its own, it may not require the formation of an endosomal retromer complex involving Snx4/Snx41/Snx42 [28]. We therefore hypothesize that MoSnx41 combines the functions (retrieval trafficking and pexophagy, respectively) of these two related sorting nexins from budding yeast. To assess functional relevance between MoSnx41 and ScSnx41/42, we expressed *ScSNX41* or *ScSNX42* in *M. oryzae snx41*Δ mutant expressing the peroxisomal GFP-SRL fusion protein [24]. 

Next, we created deletion mutants in *ATG26* or *PEX14* orthologs in *M. oryzae*. An *atg26*Δ strain [19] was created by replacing *MGG_03459* with the hygromycin-resistance cassette (*HPH1*) in the GFP-SRL strain of *M. oryzae*. *M. oryzae* Peroxin14 (Pex14) is similar to the *H. polymorpha* ortholog, which contains a central region composed of a short hydrophobic loop, an SH3 domain and a coiled-coil domain essential for its function as the peroxisomal translocon [23]. The N- and C-terminus of *M. oryzae* Pex14 (hereafter called Pex14 unless specified otherwise), both predicted to be cytosolic, are rich in Serine and Threonine residues that are potential phosphorylation sites and predicted to act as a “switch” for pexophagy [23] (Figure 2D). The *pex14*Δ mutant was generated in *M. oryzae* (*MGG_01028.6*; in GFP-SRL background). Two truncated versions of *PEX14, PEX14*_*61-361*_ and *PEX14*_*1-258*_ (Figure 2D), were individually expressed in the *pex14*Δ mutant under the native *PEX14* promoter.

**Figure 3:**
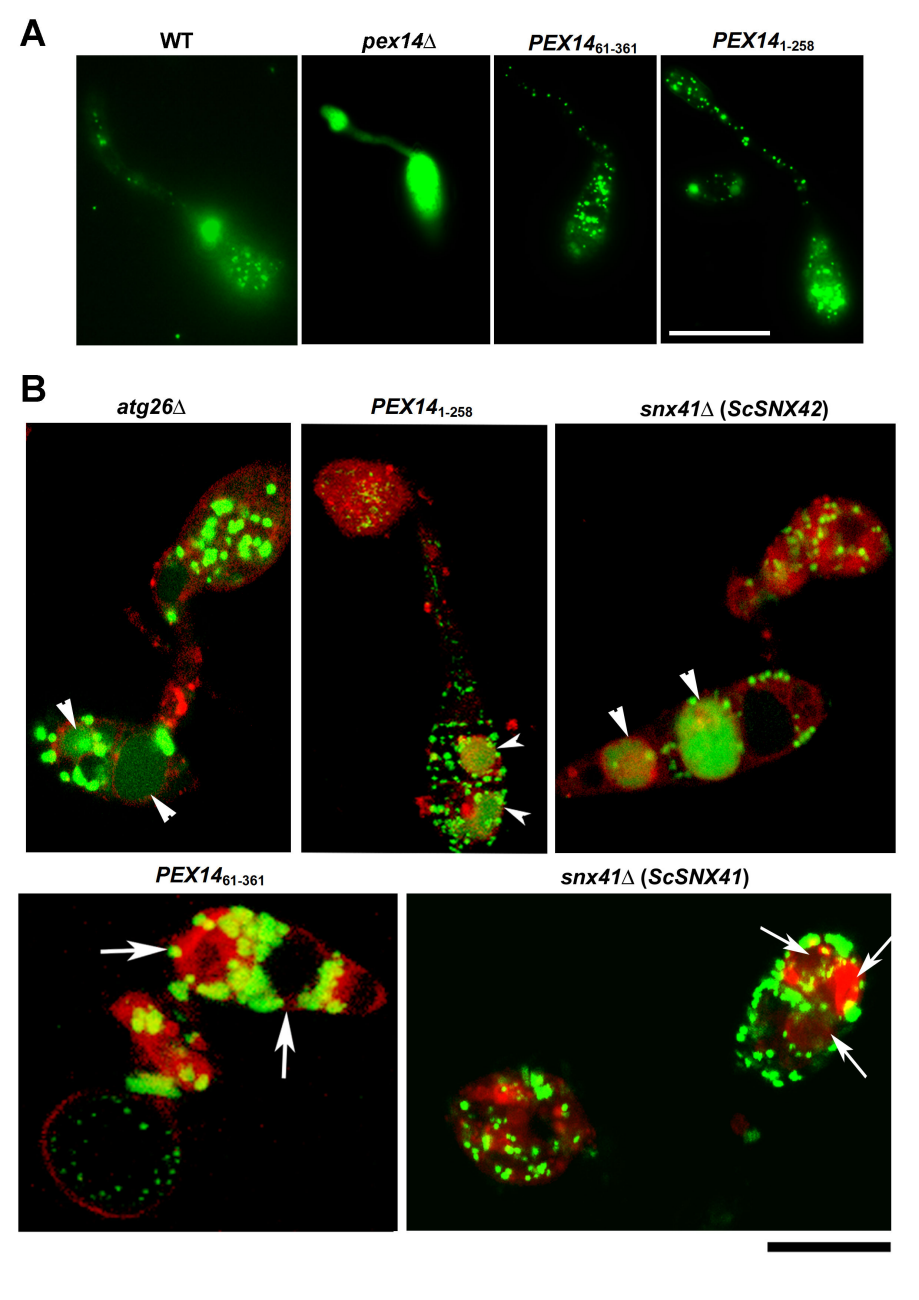
Characterization of pexophagy-deficient mutants in *M. oryzae*. (A) GFP-SRL mis-localizes to the cytoplasm in the *pex14*Δ mutant. Scale bar equals 10 micron. (B) Pexophagy is normal (restored) in *M. oryzae* *atg26*Δ, *PEX14*_*1-258*_, and *snx41*Δ (*ScSNX42*) strains (in GFP-SRL background). Vacuolar staining with Lysotracker Red DND99 was performed 5 min before confocal microscopy. Arrowheads denote vacuolar GFP-SRL. Conidia of *PEX14*_*61-361*_ and *snx41*Δ with *ScSNX41* (all in GFP-SRL background) co-stained with Lysotracker Red DND99 were analyzed by confocal microscopy. Arrows denote vacuolar compartments without GFP-SRL, indicative of a block in pexophagy. Bar = 10 μm.

As expected, GFP-SRL mis-localized to the cytoplasm in the *pex14*Δmutant (Figure 3A), likely due to loss of the predicted peroxisomal translocon function of Pex14. Expression of*PEX14*_*61-361*_ or *PEX14*_*1-258*_ as the sole copy in the deletion mutant could restore the punctate localization of GFP-SRL (Figure 3A), likely due to restoration of peroxisome biogenesis and/or peroxisomal import function of Pex14 via these two truncated Pex14 variants. 

Pexophagy was examined in the *atg26*Δ mutant, *Pex14* truncated mutants, and *ScSNX41* or *ScSNX42* complemented *snx41*Δ mutants, at the developmental stages when pexophagy is expected to occur in the wild type. Percentage (Mean ± S.E.) of vacuoles containing GFP-SRL out of total vacuoles was quantified with aforementioned mutant strains and calculated with three repeats (n>50 for each instance). Vacuolar accumulation of the GFP-SRL signal, indicative of pexophagy and similar to WT, was evident in the *atg26*Δ mutant (72.2 ± 5.6 %, *p* = 0.54 vs WT), *PEX14*_*1-258*_ mutant (69.2 ± 4.7 %, *p* = 0.27 vs WT) and *ScSNX42* complemented *snx41*Δ strain (57.4 ± 4.9 %, *p* = 0.15 vs WT) (Figure 3B, arrowheads). In contrast, numerous GFP-SRL labeled peroxisomes were observed outside the vacuoles in conidia of *PEX14*_61-361_ mutant (10.0 ± 5.8 % vacuoles containing GFP-SRL, *p* < 0.01 vs WT) and *ScSNX41*-expressing *snx41*Δ(4.8 ± 4.8 % vacuoles containing GFP-SRL, *p* <0.01 vs WT), even at the late stages of appressorium development (16-20 hpi; Figure 3B, arrows), indicating a significant reduction in or a total loss of pexophagy in the *PEX14*_61-361_ mutant and the *ScSNX41*-expressing *snx41*Δ. 

**Figure 4:**
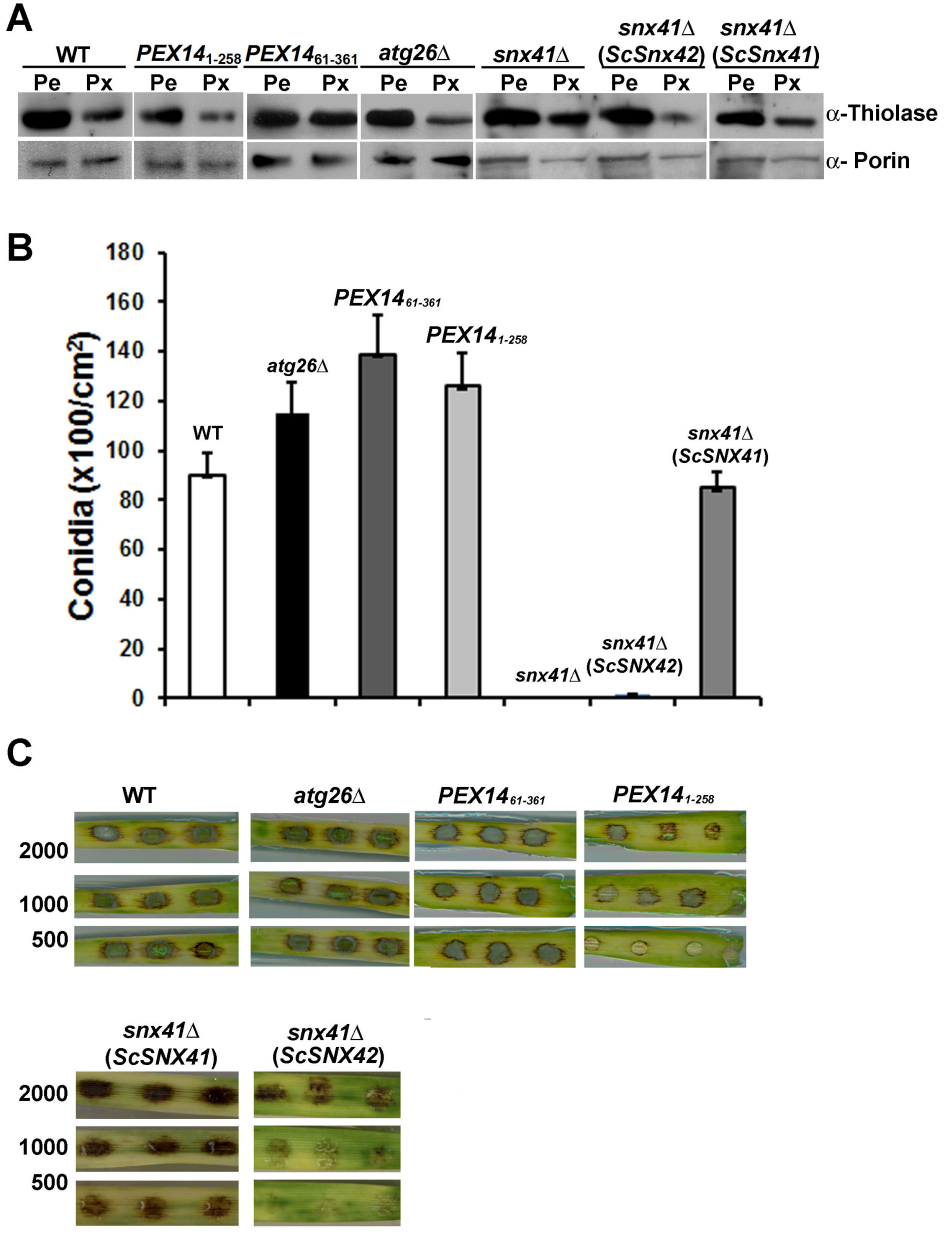
Characterization of conidiation and pathogenicity in pexophagy-deficient strains. (A) Detection of pexophagy defects by biochemical assay. WT, *atg26*Δ, *PEX14*_*1-258*_, *PEX14*_61-361_, *snx41*Δ and the two complemented strains were grown in peroxisome biogenesis (Pe) or pexophagy induction (Px) conditions. The total lysates from aforementioned strains were subjected to immunoblotting with anti-thiolase (Thi) antibody. The immunoblot was reprobed with anti-Porin antisera as loading control. Percentage denotes reduction of Thiolase in Px condition compared to the Pe condition, as an indicator of pexophagy efficiency. Percentage reduction was calculated using ImageJ [38] as follows: Percentage reduction of Thiolase level = (Density_Pe_-Density_Px_)/Density_Pe_. (B) Bar chart depicting quantification of conidiation in the wild type (WT), *atg26*Δ, *PEX14*_*61-361*_, *PEX141*_*-258*_, *snx41*Δ, *snx41*Δ (*ScSNX41*) and *snx41*Δ (*ScSNX42*) strains grown on PA medium containing lactose as the sole carbon source. Note that values present averages (±S.E.) from three independent experiments (n = 30 colonies for each sample). Total conidia counts were performed 5 d post photo-induction. (C) Barley leaf explants were spot inoculated with conidia (2,000, 1000, and 500 per droplet) from wild type (WT), *atg26*Δ, *PEX14*_*61-361*_, *PEX141*_*-258*_, *snx41*Δ (*ScSNX41*) and *snx41*Δ (*ScSNX42*) strains. Disease symptoms were assessed 7 d post inoculation.

Pexophagy was further verified by immunoblot assay using anti-Thiolase antibody [29] (*S. cerevisiae* peroxisomal thiolase, encoded by POT1 [30]) in the aforementioned strains. Wild type, *atg26*Δ, *PEX14*_61-361_ and *PEX14*_1-258_ strains, as well as *snx41*Δ mutant and the two aforementioned complemented strains, respectively, were first grown in Pe (inductive for peroxisome biogenesis: Basal Medium+olive oil) condition for 6 h, and then shifted to Px (inducing pexophagy: Complete Medium with glucose) conditions [16] for growth for another 12-16 h. As expected, Thiolase accumulated in the WT in the presence of fatty acids as the sole carbon source, but was significantly reduced (by 52 ± 7%) when grown in glucose-containing CM for 12-16 h (Figure 4A). In contrast, *snx41*Δ showed less reduction (only by 12 ± 5%, *p* = 0.01) of Thiolase during growth in pexophagy-inducing condition (Figure 4A). Thiolase clearance was comparable to the wild type in the *atg26*Δ (53 ± 14% reduction, *p* = 0.98) and *PEX14*_*1-258*_ strain (44 ± 11% reduction, *p* = 0.55), (Figure 4A), indicating that pexophagy was likely functional in these two mutants. Pexophagy was defective in the *PEX14*_61-361_ mutant, based on accumulation of Thiolase even under pexophagy-inducing condition (17 ± 7% reduction, *p* = 0.02; Figure 4A). The efficiency of pexophagy was significantly restored in the *snx41*Δ expressing *ScSNX42* (38 ± 5%reduction, *p* = 0.17 vs WT, *p* = 0.02 vs *snx41*Δ), while only marginally compensated upon complementation with *ScSNX41* (12 ± 4% reduction, *p* = 0.01 vs WT, *p* = 0.98 vs *snx41*Δ). Our systematic biochemical and cell biological analyses thus demonstrated that Atg26 is likely not required for pexophagy, while the N-terminal cytosolic domain of Pex14 is certainly essential for pexophagy in *M. oryzae*. Lastly, ScSnx42/Atg20 but not ScSnx41, could significantly restore pexophagy in *Mosnx41*Δ mutant. 

### Pexophagy is not essential for *M. oryzae* conidiation or pathogenesis

Next, we investigated the physiological relevance of pexophagy during asexual spore formation (conidiation) and infection, using the pexophagy-deficient mutants and aforementioned *Mosnx41*Δ complemented with *ScSNX41* or *ScSNX42*/*ATG20*.

Consistent with what was reported earlier [19], the *atg26*Δ mutant was capable of proper conidiation and pathogenesis (Figure 4B and C). Despite pexophagy defects, the *PEX14*_*61-361*_ mutant was fully capable of conidiation and pathogenicity (Figure 4B and C). This indicated that pexophagy may not be essential for *M. oryzae* conidiogenesis or pathogenesis. Furthermore, pexophagy and pathogenic defects could be separated in the *snx41*Δ complemented strains: conidiation and pathogenesis were fully restored in *snx41*Δ expressing *ScSNX41* (Figure 4B and C), although pexophagy was still blocked (Figures 3B and 4A). In contrast, expression of *ScSNX42* restored pexophagy (Figures 3B and 4A) but could not restore conidiation or pathogenesis in the *snx41*Δ mutant (Figure 4B and C). We conclude that pexophagy *per se* is not essential for *M. oryzae* asexual development or pathogenicity, which likely depend on other vesicular trafficking or sorting pathway(s) that requires Snx41 function.

To conclude, conidiation and pathogenicity were normal in pexophagy-deficient mutants *PEX14*_61-361_ and *snx41*Δ (*ScSNX41*), suggesting that pexophagy may not be essential for conidia formation and/or function in *M. oryzae*.

## Discussion

Atg26 was identified as a pathogenic factor in *C. orbiculare* [15], but does not appear to be important for pathogenesis in *M. oryzae* [19]. In our study, we showed that pexophagy was not dependent on Atg26 in *M. oryzae*, which may explain the dispensability of Atg26 for *M. oryzae* pathogenesis [19]. Furthermore, our findings clearly contrast to those reported for *C. orbiculare* [15,18], where pexophagy is essential for host invasion. We found that pexophagy *per se* is dispensable for *M. oryzae* pathogenesis. 

In *M. oryzae* and *Colletotrichum* species, internal turgor pressure is produced in the appressoria to facilitate breaching of the host epidermal cell during invasion [31]. During infection-related morphogenesis, the conidia of both *M. oryzae* and *C. orbiculare* metabolize the fatty acids stored in lipid bodies through β-oxidation within peroxisomes [13,14,31]. This explains why peroxisome biogenesis and its functional integrity are important for virulence, in these two fungal pathogens. It remains unclear whether the turnover of peroxisomes by pexophagy is equally important. Atg26-mediated pexophagy was shown to be essential for appressorial turgor generation, and the possible reason could be the regulation of pexophagy efficiency by removing redundant peroxisomes in the mature appressoria [15]. Such process appears less important for *M. oryzae*. Autophagic degradation of peroxisomes occurs in non-germinating conidial cells during *M. oryzae* appressorial development and maturation (Figure 1B). However, even in pexophagy-deficient mutants, the clearance/turnover of peroxisomes could be achieved at the late stage of infection (when penetration and *in planta* growth occur), probably as a consequence of autophagic cell death (ACD) in the conidial cells. ACD is essential for appressorial maturation and function [32], and likely depends on non-selective autophagy, but not on pexophagy and/or other selective subtypes of autophagy in *M. oryzae* [33]. 

We also found that Pex14 alone carries out distinct functions in both peroxisome biogenesis and turnover. As a peroxisomal membrane protein, its central region anchors Pex14 on peroxisomes and meanwhile essential for peroxisome biogenesis, by facilitating peroxisomal protein import [34,35] (Figure 2D). The N-terminal cytosolic region of Pex14 is specifically essential for pexophagy, as shown in our study (Figure 3B). It was reported recently that N-terminus of Pex14 is also required for peroxisome motility in mammalian cells [36]. Our preliminary observations suggest that it is not the case in *M. oryzae* (data not shown). 

The sorting nexin Snx41 was recently shown to be essential for *M. oryzae* conidiation and pathogenesis, mainly due to its function in retrieval trafficking of a critical enzyme involved in amino acid metabolism and/or anti-oxidant production [24]. Our previous study revealed that Snx41 was also required for pexophagy [24], but its relevance to *M. oryzae* pathogenic development was not addressed. In this study, by genetic complementation we demonstrated that retrieval trafficking and pexophagy could be independently carried out by ScSnx41 and ScSnx42, two yeast orthologs related in sequence to *M. oryzae* Snx41. We further proved that the pexophagy function of Snx41, which was restored by ScSnx42 in *snx41*Δ mutant, was dispensable for conidiation or pathogenesis. Overall, our study dissects the specific roles of Snx41 in *M. oryzae* and represents a systematic analysis of pexophagy and its biological relevance in the rice blast pathogen. 

## Materials and Methods

### Fungal strains, growth conditions and infection assay

*M. oryzae* wild-type strain B157 (Field isolate, *mat1-1*) was from the Directorate of Rice Research (Hyderabad, India). *M. oryzae* strains were propagated on Prune-agar (PA) medium or Complete medium (CM) as described [14,37]. To induce peroxisome biogenesis or pexophagy, vegetative hyphae of respective strains grown in CM liquid medium for 3 d, were shifted to basal medium containing olive oil as the sole carbon source (BM+OL; for peroxisome biogenesis), and then were shifted back to CM (to induce pexophagy). Isolation of nucleic acids, total protein extraction, quantitative analysis of conidiation, and barley leaf explants infection follow the procedure as described [25]. 

### Generation of deletion mutants

For gene deletion of *ATG26* or *PEX14*, genomic DNA fragments (about 1 kb each) representing the 5' and 3' UTR of each gene were amplified by PCR, ligated sequentially so as to flank the *HPH1* cassette. A cDNA encoding *PEX14*_*61-361*_ was generated with AMV reversed transcriptase (Roche, Cat. No. 11495062001) and amplified by PCR, and expressed with *PEX14* promoter and TrpC terminator, as a single copy in *pex14*Δ, with *ILV1* cassette as fungal selection marker. Genomic DNA encoding *PEX14*_*1-258*_ was amplified by PCR and expressed with *PEX14* promoter and TrpC terminator, as a single copy in *pex14*Δ and with the *ILV1* cassette as fungal selection marker. *ScSNX41* or *ScSNX42* coding sequence was PCR amplified and expressed with *MPG1* promoter, in the plasmid with *ILV1* cassette as fungal selection marker. The complementation construct was transformed into the GFP-SRL expressing *snx41*Δ mutant. The primers used for gene deletion or complementation are listed in Table 1. Underlined text represents the restriction enzyme site introduced for cloning purposes. 

**Table 1:** Oligonucleotide primers used in this study.

**Gene (Locus)**	**Description**	**Enzyme sites**	**Primer sequence**
*ATG26 (MGG__03459.6)*	Deletion construct	*Eco*RI	5’- GAGTGAGAATTCCAAAGCTTATCAGCTTAAGCCT-3’
		*Xma*I	5’-GAGAGTGACCCGGGTGTGGGGGAGTGTGCGCCGTTG-3’
			5’-GTTGTTCACTGCAGACCTGGCAGCC-3’
		*Hind*III	5’-GAGTGTTAAGCTTTCACACTTCCCGACCAGCGGCC-3’
*PEX14 (MGG_01028.6)*	Deletion construct	*Hind*III	5’-GAGAGTGTTAAGCTTTCGACGTGCGTTGGAAGACGGG-3’
		*Pst*I	5’-GAGACTGTTCTGCAGGTCTAGGCTAGGTCAATTACGC-3’
		*Kpn*I	5’-GAGAGTGAGGTACCACCGCACATTGGCCGTTCCGAT-3’
		*Eco*RI	5’-GAGTGAGAATTCCCGCCCGCCCTGTGACAATG-3’
	cDNA encoding AA61-361	*Pst*I	5’-GAGTGACTGCAGCAACAAGATCTTGTTCTCATATCAGCACAAAGAATGAGTGATAGAGAATAATTCAGATTAATCTGCCGCGGTATGCCGGCCGCTTACTCGGCACCAC-3’
		*Bam*HI	5’-GAGTGAGGATCCCTAGCTTGACGCAGGCGGGGTTGCT-3’
	genomic DNA encoding AA1-258	*Pst*I	5’-GAGTGACTGCAGCAACAAGATCTTGTTTCTCATATCAG-3’
		*Bam*HI	5’-GAGAGTGAGGATCCCTATGGTGTGGCGGGTCCAGTCA-3’
*ScSNX41*	genomic DNA	*Bsp*HI	5’- GAGTGATCATGATGGACTACAACATATTTGAGGCGG-3’
		*Bam*HI	5’-GAGTGAGGATCCTTATAGGGCCTCGTCGATTTGT-3
*ScSNX42*	genomic DNA	*Nco*I	5’-GAGTGACCATGGGTTCAGACTTAAATGATGTCCAAG-3’
		*Mlu*I	5’-GAGTGAACGCGTCTATGCAAAATCTTGATGTCTTTTGA-3

### Microscopy and image processing

GFP epifluorescence was observed using a Zeiss LSM510 inverted confocal microscope (Carl Zeiss Inc.) equipped with a 30 mW Argon laser. The objective used was a 100 x Achromat (n.a. 1.25) oil immersion lens. EGFP was imaged with 488 nm wavelength laser excitation, using a 505-530 nm band pass emission filter or a 505 nm long-pass emission filter, while RFP imaging used 543 nm laser and a 560 nm long-pass emission filter.

For quantitative analysis of pexophagy based on fluorescent images, we calculated the percentage of vacuoles containing GFP-SRL out of total vacuoles, and the number represents Mean ± S.E. for three repeats for each strain (n>50). Densitometry analysis with the western blotting was performed with ImageJ, following the procedure as described [38]. Percentage reduction of Thiolase was calculated with three repeats for each strain and the number represents Mean ± S.E..

### Biochemistry assays

Total protein extractions follow the reported procedure [24,25]. Protein samples from each extract (100 mg) were fractionated by SDS-PAGE, transferred onto nitrocellulose membrane (Millipore) and immunoblotted with appropriate primary antibody (anti-Thiolase, a gift from Suresh Subramani’s lab, generated against ScPot1; or anti-Porin: Invitrogen, Cat. No.459500) at the recommended dilution. Secondary antibody conjugated to horse-radish peroxidase was used at 1:20000 dilution. The Amersham ECL™ Kit (GE, Cat. No. RPN2135) was used to detect the chemiluminescent signals in Western blotting experiments.
